# Predicting Antimicrobial Resistance Prevalence and Incidence from Indicators of Antimicrobial Use: What Is the Most Accurate Indicator for Surveillance in Intensive Care Units?

**DOI:** 10.1371/journal.pone.0145088

**Published:** 2015-12-28

**Authors:** Élise Fortin, Robert W. Platt, Patricia S. Fontela, David L. Buckeridge, Caroline Quach

**Affiliations:** 1 Department of Epidemiology, Biostatistics and Occupational Health, McGill University, Montréal, Québec, Canada; 2 Direction des risques biologiques et de la santé au travail, Institut national de santé publique du Québec, Québec and Montréal, Québec, Canada; 3 Department of Pediatrics, The Montréal Children's Hospital, McGill University, Montréal, Québec, Canada; Ross University School of Veterinary Medicine, SAINT KITTS AND NEVIS

## Abstract

**Objective:**

The optimal way to measure antimicrobial use in hospital populations, as a complement to surveillance of resistance is still unclear. Using respiratory isolates and antimicrobial prescriptions of nine intensive care units (ICUs), this study aimed to identify the indicator of antimicrobial use that predicted prevalence and incidence rates of resistance with the best accuracy.

**Methods:**

Retrospective cohort study including all patients admitted to three neonatal (NICU), two pediatric (PICU) and four adult ICUs between April 2006 and March 2010. Ten different resistance / antimicrobial use combinations were studied. After adjustment for ICU type, indicators of antimicrobial use were successively tested in regression models, to predict resistance prevalence and incidence rates, per 4-week time period, per ICU. Binomial regression and Poisson regression were used to model prevalence and incidence rates, respectively. Multiplicative and additive models were tested, as well as no time lag and a one 4-week-period time lag. For each model, the mean absolute error (MAE) in prediction of resistance was computed. The most accurate indicator was compared to other indicators using t-tests.

**Results:**

Results for all indicators were equivalent, except for 1/20 scenarios studied. In this scenario, where prevalence of carbapenem-resistant *Pseudomonas* sp. was predicted with carbapenem use, recommended daily doses per 100 admissions were less accurate than courses per 100 patient-days (p = 0.0006).

**Conclusions:**

A single best indicator to predict antimicrobial resistance might not exist. Feasibility considerations such as ease of computation or potential external comparisons could be decisive in the choice of an indicator for surveillance of healthcare antimicrobial use.

## Introduction

Although the causal relationship between antimicrobial use and antimicrobial resistance is difficult to quantify due to the various settings and measures studied and to related biases, this relationship is generally accepted.[1–3] The European Surveillance of Antimicrobial Comsumption (ESAC) has shown that countries using antimicrobials more intensively tend to also present higher levels of resistance.[4] Considering that antimicrobial use is modifiable, surveillance of antimicrobial use is often recommended as a complement to surveillance of antimicrobial resistance in hospitals.[5–8]

In practice however, methodologies vary between networks and research teams. For surveillance of antimicrobial use, the World Health Organization recommends the use of defined daily doses (DDDs) per patient-days.[9] ESAC also measures hospital antimicrobial use in point prevalence surveys (proportion of patients receiving treatment), while the American National Healthcare Safety Network prefers agent-days (days of therapy [DOT]) per patient-days, among others.[6, 10, 11] Authors have suggested that the solution might reside in the monitoring of sets of indicators, but composition of these sets also varies: DDD and locally defined daily doses per patient-days[12]; daily doses per admissions and per patient-days[13, 14]; DOT, length of therapy (LOT) and the DOT:LOT ratio[15]. Although many authors have exposed either the limitations of different indicators, their own choice of indicator, or the necessity for more research to identify the most appropriate indicator(s) for surveillance of antimicrobial use, ultimately, very few published studies have actually compared these indicators’ ability to predict resistance levels in hospital settings.[1, 11, 15–18]

Public health authorities or hospital epidemiologists wishing to develop a coordinated program devoted to the surveillance of hospital antimicrobial use have to identify one or a few of these indicators for their surveillance. However, the optimal way to measure antimicrobial use in hospital populations, to complement surveillance of resistance, is still unclear. Using respiratory isolates and antimicrobial prescriptions of nine intensive care units, and assuming a causal association between antimicrobial use and resistance, this study thus aimed to identify the indicator of antimicrobial use that predicted prevalence and incidence rates of resistance in ICUs with the best accuracy. Specifically, the objective was not, however, to demonstrate the existence of a causal association between antimicrobial use and resistance, nor was it to quantify such an association without bias.

## Methods

This study was approved by the Research Ethics Boards of McGill University (study number A10-M105-10B) and of the *Centre Hospitalier Universitaire* Sainte-Justine (study number 3305). No consent from patients was necessary as the data was analyzed anonymously.

### Study design and population

This was a retrospective cohort study on all patients admitted to ICUs of four hospitals located in Montreal, Canada, between April 1^st^, 2006 and March 31^st^, 2010. Participating ICUs included three neonatal ICUs (NICU), two pediatric ICUs (PICU) and four adult ICUs. The study design focused on recreating a surveillance context to identify the best indicator of antimicrobial use for surveillance activities, in this case, an ICU-based surveillance.

Admission–Discharge–Transfer (ADT) data were extracted for all ICU patients. Our data included unique identifying numbers, ICU identification and dates of admission and discharge to and from hospitals and ICUs. After a careful review, these data were used to compute numbers of ICU admissions (including transfers from other wards), numbers of patients present in the ICU and ICU patient-days, per 4-week period, for each ICU.[19]

### Antimicrobial resistance

Using microbiology laboratory information systems, a database of bacteria isolated from ICU patients was built. Variables extracted were: unique identifying number, ICU identification, sampling site, sample collection date, identified microorganism, antimicrobial tested and resistance profile (susceptible, intermediate or resistant). This database was merged with ADT data, to link cultures with specific care episodes. Susceptibility tests performed on positive respiratory tract cultures were selected. We assumed that a large proportion of ICU patients were intubated at some point during their ICU stay and that respiratory cultures were done for intubated patients as part of the investigation for unstable ICU patients. This was thus an attempt to describe the respiratory microbiota, regardless of the presence of an infection. Intermediate strains were counted with susceptible as non-resistant strains. For the measurement of resistance prevalence and incidence, clinically relevant antimicrobial resistances (microorganism / antimicrobial combinations) were selected. Coliforms were analyzed as a group and included the following microorganisms: *Enterobacter* sp., *Escherichia coli*, *Hafnia alvei*, *Klebsiella* sp., *Morganella morganii*, *Providencia rettgeri*, *Raoultella* sp., *Serratia* sp. and microorganisms coded as “Coliforms”.

Based on the Society for Healthcare Epidemiology of America (SHEA) and Healthcare Infection Control Practices Advisory Committee (HICPAC) recommendations for metrics for multidrug-resistant organisms in healthcare settings, prevalence of resistance per 100 ICU admissions was measured to estimate exposure burden and incidence of resistance per 10,000 patient-days was also measured to quantify healthcare acquisition.[20] Prevalence of resistance was measured by counting the number of ICU admissions where a resistant strain of a given microorganism was isolated. Resistance was considered incident when a resistant microorganism was detected in a patient with a previously susceptible organism or with no positive culture at least 2 days after admission to ICU; patient-days were computed excluding the first 2 days after ICU admission, based on dates, as these patient-days had an event probability equal to zero. Incidence rates and prevalence were computed per 4-week period, for each ICU.

### Antimicrobial use

Hospital pharmacy databases provided information on all prescriptions for antimicrobials issued for patients included in the study: unique identifying number, age, weight, antimicrobial prescribed, posology and prescription start and stop dates. These databases described prescribed drugs and not necessarily drugs administered to a patient. Only agents belonging to class J01 of the Anatomical Therapeutic Chemical (ATC) classification system (anti-infectives for systemic use) were kept for analysis.[21] Doses and days of treatment prescribed for use before or after ICU admission were excluded (as these would not be included in an ICU-based surveillance), but we included those used on the ICU admission or discharge dates, or in between; in contrast with resistance computations, the first two days of each stay were kept in measurements of antimicrobial use).

Population antimicrobial use was measured using fifteen different indicators. These indicators were obtained by combining five numerators (defined daily doses [DDDs], recommended daily doses [RDDs], agent-days, exposed patients, and number of courses) with three denominators (ICU patient-days, ICU admissions and ICU patients), all previously identified in a systematic review of indicators used for populations that included pediatric patients.[11] DDDs were computed dividing quantities prescribed by the standard values specified on the website of the ATC/DDD system.[21] RDD were computed similarly, but standard values for the computation of RDDs were based on doses recommended in the 2008 Sanford Guide, the 2012 Red Book, The Montreal Children’s Hospital drug formulary and Nelson 2012; pediatric patients’ weight was accounted for in RDD computations (RDDs in mg/kg).[22–25] A table of standard values is provided as supporting information (S1 Table). Agent-days were the numbers of days when each specific antimicrobial was prescribed; in a combined therapy, each agent prescribed for a day counts for one agent-day. Exposure was the number of patients prescribed an antimicrobial agent, regardless of quantity or duration. Courses were the number of distinct periods of consecutive days when a patient was prescribed a specific antimicrobial. All ICU patient-days were included in the computation of denominators, as antimicrobial exposure was measured for the entire ICU stay; ICU admission and discharge day each counted for half a day. For a given 4-week period, “ICU admissions” only include patients admitted to the ICU during the period, while “ICU patients” include all patients present in the ICU at some point in time during the period. Indicators of antimicrobial use were computed per 4-week period, for each ICU.

Ten different resistance / antimicrobial use combinations were studied and are listed in the first column of Tables 1 and 2, for a total of 20 scenarios: 10 for prediction of prevalence and 10 for prediction of incidence rates. These combinations were selected based on the frequency of resistant strains in the Province of Quebec, and on their clinical relevance. In two combinations, use of three classes of antimicrobials was taken into account, as *Staphylococcus aureus* resistant to methicillin and *Escherichia coli*, *Klebsiella* sp. and *Proteus* sp. resistant to carbapenems can simultaneously present other resistances. All of these additional antimicrobials can thus also indirectly contribute to selection of targeted resistances.

**Table 1:** Most accurate, second most accurate and least accurate indicators in predicting resistance prevalence, for selected resistance /antimicrobial combinations.

Resistance / antimicrobial use*	Models adjusted for ICU type	Most accurate			Second most accurate				Least accurate			
		Indicator	Regression link	MAE (cases / 100 adm)	Indicator	Regression link	MAE (cases / 100 adm)	Difference (p-value)**	Indicator	Regression link	MAE (cases / 100 adm)	Difference (p-value)***
MRSA / penicillins	NICU, Others (ref.)	DDD / patients	Id	0.55	DDD / adm	Id	0.55	1.00	Agent-days / adm	Log	0.58	0.52
MRSA/ penicillins + 3GC + quinolones	NICU, Others (ref.)	DDD / adm	Log	0.53	DDD / patients	Log	0.53	0.94	Courses / pd	Log	0.58	0.29
Pip-tazo-resistant coliforms / pip-tazo	Unadjusted	DDD / adm	Id	0.62	RDD / patients	Id	0.62	0.99	Agent-days / adm	Log	0.70	0.33
Quinolone-resistant coliforms / quinolones	NICU, PICU, AICU (ref.)	DDD / adm (1)	Id	0.32	DDD / adm	Id	0.32	0.94	Exposed / pd (1)	Log	0.38	0.12
Aminoglycoside-resistant coliforms /aminoglycosides	Unadjusted	DDD / pd	Id	0.38	DDD / patients	Id	0.39	0.97	DDD/ adm (1)	Log	0.41	0.51
Carbapenem-resistant EKP/ carbapenems	Unadjusted	Agent-days/ pd	Id	0.21	Courses/ pd	Id	0.21	0.95	RDD / adm (1)	Log	0.24	0.36
Carbapenem-resistant EKP/ 3GC + aminoglycosides + quinolones	Unadjusted	DDD / adm	Id	0.21	DDD / patients	Id	0.21	0.97	Agent-days / adm	Id	0.26	0.30
Pip-tazo-resistant *Pseudomonas* sp. / pip-tazo	Unadjusted	DDD / adm (1)	Id	0.30	DDD / patients (1)	Id	0.31	0.95	Agent-days / adm (1)	Log	0.33	0.70
Quinolone-resistant *Pseudomonas* sp. / quinolones	Unadjusted	Courses / adm (1)	Id	0.16	Exposed / patients (1)	Id	0.16	0.98	Exposed / adm (1)	Id	0.34	0.0043
Carbapenem-resistant *Pseudomonas* sp. / carbapenems	NICU, PICU, AICU (ref.)	Courses / pd	Id	0.31	Agent-days / pd	Id	0.32	0.85	RDD / adm (1)	Log	0.43	0.0006

Note: (1): with a time lag of one 4-week period; 3GC: third-generation cephalosporins; AICU: adult intensive care unit; adm: admissions; EKP: *Escherichia coli*, *Klebsiella* sp., *Proteus* sp.; Id: identity link (additive model); Log: log link (multiplicative model); MAE: mean absolute error; MRSA: meticillin-resistant Staphylococcus aureus; NICU: neonatal intensive care unit; pd: patient-days; PICU: pediatric intensive care unit; pip-tazo: piperacillin-tazobactam.

*Resistance / antimicrobial use: “resistance” designates the resistant microorganism prevalence that was predicted using the population use of the designated”antimicrobial use”.

**Level of statistical significance, after a Holm correction for multiple comparisons: 0.05 / 1 = 0.05.

***Level of statistical significance, after a Holm correction for multiple comparisons: 0.05 / 59 = 0.0008.

**Table 2:** Most accurate, second most accurate and least accurate indicators in predicting resistance incidence rates, for selected resistance /antimicrobial combinations.

Resistance / antimicrobial use*	Model adjusted for ICU type	Most accurate			Second most accurate				Least accurate			
		Indicator	Regression link	MAE (cases / 10,000 pd)	Indicator	Regression link	MAE (cases / 10,000 pd)	Difference (p-value)**	Indicator	Regression link	MAE (cases / 10,000 pd)	Difference (p-value)***
MRSA / penicillins	NICU, PICU, AICU (ref.)	Agent-days/ patients (1)	Log	9.8	Courses/ patients (1)	Log	9.8	0.97	Courses/ pd	Id	10.4	0.50
MRSA/ penicillins + 3GC + quinolones	NICU, PICU, AICU (ref.)	Exposed/ adm (1)	Log	8.6	Exposed/ patients (1)	Log	8.6	0.97	Exposed/ pd	Id	10.4	0.03
Pip-tazo-resistant coliforms / pip-tazo	NICU, Others (ref.)	RDD/ patients (1)	Id	11.5	DDD/ patients (1)	Id	11.6	0.97	Agent-days/ adm (1)	Log	12.7	0.28
Quinolone-resistant coliforms / quinolones	NICU, PICU, AICU (ref.)	Courses/ adm	Log	6.9	Courses/ patients	Log	6.9	0.96	Exposed/ pd (1)	Log	7.6	0.32
Aminoglycoside-resistant coliforms /aminoglycosides	Unadjusted	DDD/ adm	Id	6.0	Courses/ pd	Id	6.0	0.99	Agent-days/ adm (1)	Log	6.2	0.67
Carbapenem-resistant EKP/ carbapenems	NICU, Others (ref.)	Agent-days/ pd	Id	3.5	Courses/ pd	Id	3.5	0.93	RDD/ patients (1)	Id	4.2	0.14
Carbapenem-resistant EKP/ 3GC + aminoglycosides + quinolones	NICU, Others (ref.)	DDD/ patients	Id	3.8	DDD/ adm	Id	3.8	1.00	Exposed/ pd (1)	Log	4.2	0.43
Pip-tazo-resistant *Pseudomonas* sp. / pip-tazo	NICU, Others (ref.)	Agent-days/ patients	Log	5.3	Agent-days/ adm	Log	5.4	0.93	Courses/ pd (1)	Log	5.8	0.42
Quinolone-resistant *Pseudomonas* sp. / quinolones	Unadjusted	DDD/ adm	Id	3.5	DDD/ patients	Id	3.5	0.94	Exposed/ pd	Log	4.3	0.10
Carbapenem-resistant *Pseudomonas* sp. / carbapenems	NICU, PICU, AICU (ref.)	Courses/ pd	Log	7.1	DDD/ pd	Log	7.2	0.95	Courses/ pd (1)	Log	8.0	0.21

Note: (1): with a time lag of one 4-week period; 3GC: third-generation cephalosporins; AICU: adult intensive care unit; adm: admissions; EKP: *Escherichia coli*, *Klebsiella* sp., *Proteus* sp.; Id: identity link (additive model); Log: log link (multiplicative model); MAE: mean absolute error; MRSA: meticillin-resistant Staphylococcus aureus; NICU: neonatal intensive care unit; pd: patient-days; PICU: pediatric intensive care unit; pip-tazo: piperacillin-tazobactam.

*Resistance / antimicrobial use: “resistance” designates the resistant microorganism incidence that was predicted using the population use of the designated”antimicrobial use”.

**Level of statistical significance, after a Holm correction for multiple comparisons: 0.05 / 1 = 0.05.

***Level of statistical significance, after a Holm correction for multiple comparisons: 0.05 / 59 = 0.0008.

### Statistical analyses

For each combination, scatterplots of resistance and antimicrobial use according to the different indicators were produced to visualize aggregation of data at the ICU level and time series were produced to see trends. After adjustment for ICU type, indicators of antimicrobial use were successively tested in regression models, to predict resistance prevalence and incidence rates, per 4-week time period, per ICU (data available in S2 Table). Binomial regression was used to model prevalence and Poisson regression, to model incidence rates. Multiplicative (log link) and additive (identity link) models were tested, [26] as well as no time lag and a one 4-week-period time lag; in total, for each scenario, 60 models were compared (15 indicators x 2 regression links x 2 time lags). As there were repeated measurements for every ICU (51 measurements with a 1-period time lag, 52 measurements without a time lag), generalized estimating equations were used to account for correlated values at the ICU level.

For each model, the mean absolute error (MAE) was computed. The MAE is a statistic used in the analysis of time series, to quantify the difference (or error) between observed values (prevalence or incidence rates) and values predicted by a model.[27] Predictive accuracy of different regression models can be compared using t-tests, to determine whether differences observed in predictions are statistically significant. Absolute values of these errors were computed per 4-week period and per ICU, and were then averaged, to produce the MAE of each model. The most accurate indicators were the ones with the smallest MAEs. MAEs were then compared using t-tests. In a given scenario, the most accurate model was compared to all other models (59 t-tests), beginning with the least accurate model. A Holm correction was applied to account for multiple comparisons, to keep an overall α of 0.05.[28] As an indication, when comparing the smallest MAE to the largest one, the t-test p-value had to be smaller than 0.0008 to reject the null hypothesis. Analyses were performed using SAS 9.3.

## Results

MAEs for the most, second most and least accurate indicators, for each combination, are presented in Table 1 (prevalence) and Table 2 (incidence rates). As regression models were adjusted for ICU type, MAEs stratified per ICU type are also provided in S3 and S4 Tables. The most and least accurate indicators were usually not statistically different, except in the prediction of resistance prevalence in *Pseudomonas* sp. (cut-off value of 0.0008, given the Holm correction). When using carbapenem use to predict prevalence of carbapenem-resistant *Pseudomonas* sp., the indicator with the smallest MAE was courses per 100 patient-days (no time lag, using an identity link), which was significantly more accurate (p = 0.0006) than the least accurate model, RDD per 100 admissions (with a 1-period time lag, using a log link).

In regression models, additive models (identity link) frequently failed at producing coefficients and predicting prevalence or incidence. This was the case for 40 / 600 models for prediction of resistance prevalence and for 99 / 600 models for prediction of incidence rates, while all multiplicative models (log link) converged and produced coefficients. This problem was due to the fact that predicted values below 0 or above 1 in the case of binomial regression, or simply below 0 for Poisson regression, were obtained with additive models.

**Fig 1:**
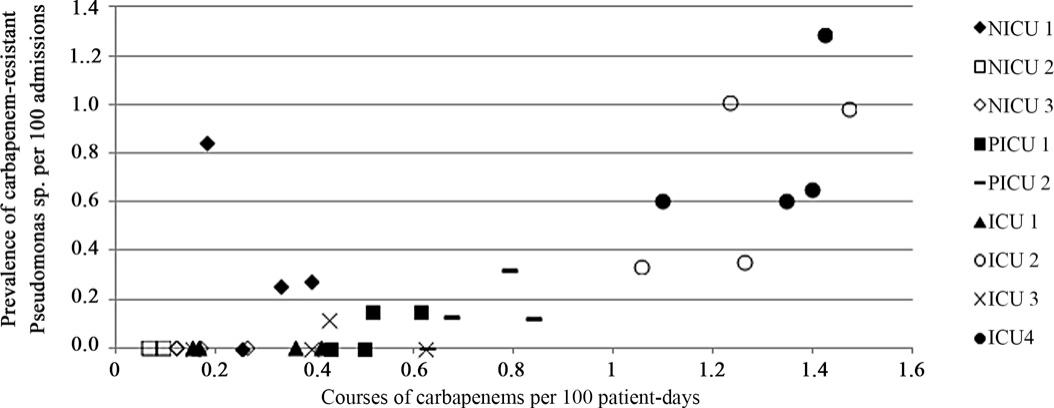
Scatterplot of prevalence of carbapenem-resistant *Pseudomonas* sp. per 100 admissions and carbapenem use in courses per 100 patient-days, per year and per intensive care unit (ICU).

**Fig 2:**
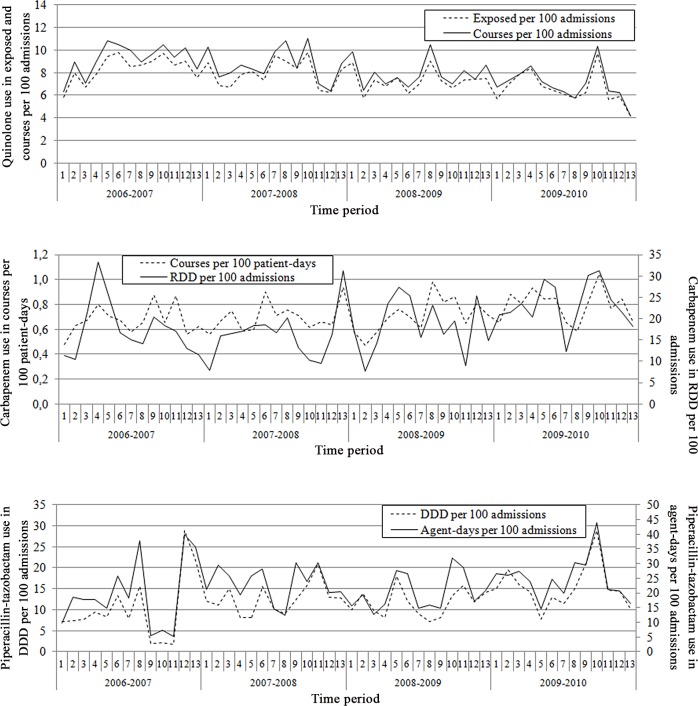
Time series of piperacillin-tazobactam, quinolone and carbapenem use per 4-week period, all ICUs combined. Part A: quinolone use in courses per 100 admissions and exposed per 100 admissions; part B: carbapenem use in courses per 100 patient-days and in RDD per 100 admissions; part C: piperacillin-tazobactam use in DDD per 100 admissions and agent-days per 100 admissions.

Examples of descriptive graphs produced are presented in Figs 1 and 2. Fig 1 presents a scatterplot of prevalence of carbapenem-resistant *Pseudomonas* sp. per 100 admissions against carbapenem use in courses per 100 patient-days, the most accurate indicator for this combination. Data was aggregated per ICU; it was also aggregated per year rather than per period, to make the graph clearer. This is representative of most scatterplots produced, showing an apparent clustering of antimicrobial use at the ICU level. Fig 2 presents time series of piperacillin-tazobactam, quinolone and carbapenem use per 4-week period, all ICUs combined. Each graph presents the most accurate and the least accurate indicators for the prediction of resistance in *Pseudomonas* sp.. As observed in MAE comparisons, indicators are visually more similar for piperacillin-tazobactam use and quinolone use than with carbapenem use, for which the only difference in predictive accuracy of resistance prevalence was observed.

## Discussion

To our knowledge, this comparison of population antimicrobial use indicators’ ability to predict resistance is novel, even though this knowledge gap had been highlighted in the scientific literature previously. Using respiratory tract isolates and antimicrobial prescriptions from nine intensive care units, this study compared the accuracy of fifteen indicators of population antimicrobial use in predicting prevalence and incidence rates of different resistances in the respiratory microbiota. A statistically significant difference between MAEs was observed for only 1 of the 20 scenarios studied: carbapenem use to predict prevalence of carbapenem-resistant *Pseudomonas* sp. This difference identified one indicator that did not perform as well; however, no single indicator (or no set of indicators) stood out as better than the others.

### Identifying the most accurate indicator

The absence of difference between indicators that was observed for most scenarios could be explained by different factors. These are not limitations, but rather reflect the reality of ICUs and of their use of antimicrobials. First, as described in Fig 1, throughout the four years of the study, levels of antimicrobial use and resistance tended to correlate at the ICU level; after adjusting for ICU type, there was thus less variation that could be explained with indicators of antimicrobial use. In addition, all indicators were attempting to measure similar variations in time: no exposure is equal to a value of zero for all numerators, which will tend to increase or decrease together with different magnitudes. Also, ICUs’ median lengths of stay are very short (5 days in neonatal ICUs and 2 days in other ICUs, data not published); for an ICU admission of two days, the difference between the number of agent-days, the number of courses and the simple exposure cannot be as large as for a longer admission. Of note, the situation would not necessarily have been dramatically different using hospital-wide data, as the median length of stay in Québec acute-care hospitals was 4 days.[29] Finally, although resistance / antimicrobial use combinations studied included entire antimicrobial classes (sometimes even three), a single agent can sometimes constitute most of an antimicrobial class usage. For instance ampicillin-days represented 67% of all penicillin agent-days; therefore, even if the standard DDD and RDD for ampicillin are very different, indicators of ampicillin use using DDDs, RDDs or agent-days will tend to follow the same time trends, and respective indicators of penicillin use will be driven by ampicillin use. In this study, rather than the actual values of indicators, what mattered were variations in time and across ICUs, and correlation of indicators with resistance measures; most indicators did not differ at these levels. Whenever a difference was found, it was between the most accurate and the least accurate indicators, but most indicators’ MAEs were not different from the indicator with the smallest MAE.

Actually, statistically different MAEs were observed only for one scenario, involving prevalence of resistance to carbapenems in *Pseudomonas* sp.. *Pseudomonas* sp. are prone to the development of resistance, especially to imipenem: they might react more swiftly to an exposure to antimicrobials, amplifying the possibility to detect differences between indicators of antimicrobial use.[30] Another interesting observation regarding this scenario is that, although a large proportion of prevalent cases are also incident cases, no statistically significant difference between indicators was detected in their prediction of incidence rates. As admissions last longer in neonatal ICUs than in other ICUs, prevalence and incidence rates do not follow the same trends, despite their similar numerators.

### Limitations

In this study, different indicators of antimicrobial use usually had similar accuracy in the prediction of resistance prevalence or incidence in the respiratory microbiota. Interpretation of results is however limited by assumptions made in the study design. In this study, we assumed that surveillance would ideally include pediatric populations, which have been frequently excluded from antimicrobial use surveillance.[15, 17, 31, 32] We also assumed that ICUs would perform surveillance on a 4-week period basis, accounting for no time lag or for a 1-period time lag, without information on antimicrobial use before ICU admission, and that neonatal, pediatric and adult ICUs would be considered different enough to be treated separately. We also limited our cohort to ICU patients and to respiratory tract cultures performed for these patients, in an attempt to include colonizing microorganisms rather than only microorganisms infecting patients. All this was done to be as representative and similar as possible to a real surveillance setting, but results could differ if other assumptions were made. Second, our results describe prescribed antimicrobials rather than administered or dispensed antimicrobials, which probably lead to some degree of overestimation of antimicrobial use in our ICUs. As we were interested in prediction of resistance time trends, this might not be as critical as for a study estimating association between antimicrobial use and resistance. Also, a study with more participating ICUs would have allowed the use of hierarchical models with random intercepts, rather than population average models using generalized estimating equations. More participating ICUs would have also allowed us to compare ICUs and perform benchmarking, but this was not our objective. Finally, with nine ICUs followed during four years, the study population was large enough to detect significant associations between some indicators and resistance levels, but a lack of power to detect differences between indicators was still possible (not enough ICU-4-week-periods observed). This possibility was however rejected after a simulation study replicating this cohort study in two much larger simulated networks of ICUs failed to demonstrate differences between indicators in a majority of studied scenarios.

## Conclusion

Indicators of population antimicrobial use have been developed, used and discussed for many decades now, but the identification of the best indicator is still an object of debate. We believe that the purpose of measurement, surveillance of antimicrobial use as a complement to surveillance of antimicrobial resistance, has to be taken into consideration. Our study has shown that, at least in our context, indicators are equivalent. Had an indicator been more accurate than others, it would have allowed a closer monitoring of variations in antimicrobial resistance frequency, and an increased ability to detect the impact on resistance of interventions targeting antimicrobial use. These first results indicate that a single best indicator might not exist and that feasibility considerations, such as ease of computation or potential external comparisons could be more decisive in the choice of an indicator for surveillance of healthcare antimicrobial use.

## Supporting Information

S1 Table Standard values used in the computation of defined daily doses (DDD) and recommended daily doses RDD). (PDF)

S2 Table Minimal dataset. (XLSX)

S3 Table Most accurate, second most accurate and least accurate indicators in predicting prevalence of antimicrobial resistance, for different scenarios, with their regression link and their mean absolute error, stratified per ICU type as adjusted for in regression models. (PDF)

S4 Table Most accurate, second most accurate and least accurate indicators in predicting incidence rates of antimicrobial resistance, for different scenarios, with their regression link and their mean absolute error, stratified per ICU type as adjusted for in regression models. (PDF)

## Abbreviations

3GC: third-generation cephalosporins
Adm: admissions
ADT: admissions–discharges—transfers
AICU: adult intensive care unit
ATC: Anatomical Therapeutic Chemical
DDD: defined daily dose
DOT: days of therapy
EKP: *Escherichia coli*, *Klebsiella* sp. and *Proteus* sp
ESAC: European Surveillance of Antimicrobial Consumption
HICPAC: Healthcare Infection Control Practices Advisory Committee
ICU: intensive care unit
LOT: length of therapy
MAE: mean absolute error
MRSA: methicillin-resistant *Staphylococcus aureus*
NICU: neonatal intensive care unit
Pd: patient-days
PICU: pediatric intensive care unit
Pip-tazo: piperacillin-tazobactam
RDD: recommended daily doses
SHEA: Society for Healthcare Epidemiology of America
