# Supplementary material for: Predicting Antimicrobial Resistance Prevalence and Incidence from Indicators of Antimicrobial Use: What Is the Most Accurate Indicator for Surveillance in Intensive Care Units?
Source: PLoS One. 2015 Dec 28;10(12):e0145088. doi: 10.1371/journal.pone.0145088 (PMC4692550; doi:10.1371/journal.pone.0145088)
Supplement: S1 Table — (PDF) [file pone.0145088.s001.pdf]

Table. Standard values used in the computation of defined daily doses (DDD) and recommended daily doses RDD).

			DDD (in mg)	RDD (in mg)							
						Neonates					
						0 - 7 days old			8 - 28 days old		
Antimicrobial class	Antimicrobial agent	Route	All patients	Adult patients	Pediatric patients	< 1.2 kg	1.2 - 2.0 kg	≥ 2.0 kg	< 1.2 kg	1.2 - 2.0 kg	≥ 2.0 kg
Tetracyclines	Demeclocycline	PO	600	600							
	Doxycycline	PO	100	150	3						
		IV	100								
	Minocycline	PO	200	200							
	Tetracycline	PO	1000	1000	37,5						
	Tigecycline	IV	100	100							
Penicillins	Amoxicillin	PO	1000	1125	90						30
	Ampicillin	IV	2000	7200	200	100	100	150	150	150	200
	Cloxacillin	PO	2000	3000	100	75	75	112,5	75	112,5	150
		IV	2000	6000	150	75	75	112,5	75	112,5	150
	Penicillin G	IV	3600	12000	150	60	60	90	60	90	120
	Penicillin V	PO	2000	1300	37,5						
	Piperacillin	IV	14000	16800	400	100	100	200	200	200	300
Penicillins (and enzyme inhibitors)	Amioxicillin-clavulanate	PO	1000	1125	90			30			30
	Piperacillin-tazobactam	IV	14000	12000	400	100	100	200	200	200	300
	Ticarcillin-clavulanate	IV	15000	14400	300	150	150	225	225	225	300
Cephalosporins	Cefazolin	IV	3000	2600	75	40	40	40	40	40	60
	Cephalexin	PO	2000	2600	37,5						
	Cefaclor	PO	1000		30						
	Cefoxitin	IV	6000	6000	120						40
	Cefprozil	PO	1000	750	22,5	22,5	22,5	22,5	22,5	22,5	22,5
	Cefuroxime	IV	3000	4125	150	100	100	150	150	150	150
		PO	500	1500	25						
	Cefepime	IV	2000	3600	100						
Cephalosporins (3rd generation)	Cefixime	PO	400	400	8						
	Cefotaxime	IV	4000	5150	150	100	100	150	100	150	150
	Ceftazidime	IV	4000	3750	150	100	100	100	150	150	150
	Ceftriaxone	IV	2000	1875	100	50	50	50	50	50	75
Monobactams	Aztreonam	IV	4000	8000	120	60	60	90	90	90	120
Carbapenems	Ertapenem	IV	1000	1000	30						
	Imipenem-cilastatin	IV	2000	2500	80	50	50	50	50	75	75
	Meropenem	IV	2000	4000	60	40	40	40	60	60	60
Sulfonamides and trimethoprim	Sulfadiazine	PO	600	3000	100	100	100	100	100	100	100
	Trimethoprim-sulfamethoxazole	PO	1200	1600	10	1,2	1,2	1,2	1,2	1,2	1,2
		IV	5500	5250	15	1,2	1,2	1,2	1,2	1,2	1,2
	Trimethoprim	PO	400	200	10	1,2	1,2	1,2	1,2	1,2	1,2
		IV	400		10	1,2	1,2	1,2	1,2	1,2	1,2
Macrolides	Azithromycin	PO	300	250	5	5	5	5	10	10	10
		IV	500	250	5	5	5	5	10	10	10
	Clarithromycin	PO	500	750	15						
	Erythromycin	PO	1000	1125	40	20	20	20	30	30	30
		IV	1000	1500	40	20	20	20	30	30	30
Lincosamides	Clindamycin	PO	1200	775	25	10	10	15	10	15	15
		IV	1800	2000	30	10	10	15	15	15	20
Aminoglycosides	Amikacin	IV	1000	1050	30	10	15	20	10	22,5	30
	Gentamicin	IV	240	350	7,5	2,85	5	5	5	5	5
		PO				10	10	10	10	10	10
	Streptomycin	IV	1000	1050							
	Tobramycin	In	300	600	600						
		IV	240	350	7,5	2,85	5	5	5	5	5
Quinolones	Ciprofloxacin	PO	1000	1000	30	30	30	30	30	30	30
		IV	500	720	30	30	30	30	30	30	30
	Levofloxacin	PO	500	500	16	16	16	16	16	16	16
		IV	500	500	16	16	16	16	16	16	16
	Moxifloxacin	PO	400	400							
		IV	400	400							
Glycopeptides	Vancomycin	IV	2000	2000	40	25	25	36	30	30	44
		PO	2000	1250	40	40	40	40	40	40	40
		Re		2000							
Amphenicols (grouped with "others")	Chloramphenicol	IV	3000	4000	75	25	25	25	25	25	30
Streptogramins (grouped with "others")	Quinupristin-dalfopristin	IV	1500	1575	22,5						
Others	Colistimethate	In	240	160							
		IV	240	175							
	Daptomycin	IV	280	280	7	12	12	12	12	12	12
	Fusidate	PO	1500	1500							
	Linezolid	PO	1200	1200	30	20	20	30	30	30	30
		IV	1200	1200	30	20	20	30	30	30	30
	Metronidazole	PO	2000	1500	30	7,5	7,5	15	15	15	30
		IV	1500	1500	30	7,5	7,5	15	15	15	30
	Nitrofurantoin	PO	200	300	6						
