# Supplementary material for: Predicting Antimicrobial Resistance Prevalence and Incidence from Indicators of Antimicrobial Use: What Is the Most Accurate Indicator for Surveillance in Intensive Care Units?
Source: PLoS One. 2015 Dec 28;10(12):e0145088. doi: 10.1371/journal.pone.0145088 (PMC4692550; doi:10.1371/journal.pone.0145088)
Supplement: S3 Table — (PDF) [file pone.0145088.s003.pdf]

S1 Table. Most accurate, second most accurate and least accurate indicators in predicting prevalence of antimicrobial resistance, for different scenarios, with their regression link and their mean absolute error, stratified per ICU type as adjusted for in regression models.

Resistance / antimicrobial use	Adjustment for ICU type	Most accurate indicator			Second most accurate indicator			Least accurate indicator		
		Indicator	Regression link	MAE	Indicator	Regression link	MAE	Indicator	Regression link	MAE
ARC / aminoglycosides	none	DDD / pd	identity	0,0038393	DDD / patients	identity	0,0038565	DDD / adm (1)	log	0,0041023
CREKP / 3GC + aminoglycosides + quinolones	none	DDD / adm	identity	0,0021132	DDD / patients	identity	0,0021225	agent-days / adm	identity	0,0026038
CREKP / carbapenems	none	agent-days / pd	identity	0,0021191	courses / pd	identity	0,0021344	RDD / adm (1)	log	0,0023601
CRP / carbapenems	adult	courses / pd	identity	0,0045931	exposed / pd	identity	0,0047914	RDD / adm (1)	log	0,0050144
	pediatric	courses / pd	identity	0,0026350	exposed / pd	identity	0,0024055	RDD / adm (1)	log	0,0019548
	neonatal	courses / pd	identity	0,0015013	exposed / pd	identity	0,0014481	RDD / adm (1)	log	0,0050144
MRSA / 3GC + penicillins + quinolones	adult and pediatric	DDD / adm	log	0,0071347	DDD / patients	log	0,0071883	courses / pd	log	0,0078887
	neonatal	DDD / adm	log	0,0016708	DDD / patients	log	0,0016594	courses / pd	log	0,0016154
MRSA / penicillins	adult and pediatric	DDD / patients	identity	0,0075407	DDD / adm	identity	0,0075353	agent-days / adm	log	0,0078824
	neonatal	DDD / patients	identity	0,0014381	DDD / adm	identity	0,0014523	agent-days / adm	log	0,0016383
PTRC / piperacillin-tazobactam	none	DDD / adm	identity	0,0062378	RDD / patients	identity	0,0062450	agent-days / adm	log	0,0070138
PTRP / piperacillin-tazobactam	none	DDD / adm (1)	identity	0,0030336	DDD / patients (1)	identity	0,0030554	agent-days / adm	log	0,0032751
QRC / quinolones	adult	DDD / adm (1)	identity	0,0051602	DDD / adm	identity	0,0049975	exposed / pd (1)	log	0,0063517
	pediatric	DDD / adm (1)	identity	0,0010252	DDD / adm	identity	0,0014714	exposed / pd (1)	log	0,0008862
	neonatal	DDD / adm (1)	identity	0,0020517	DDD / adm	identity	0,0020339	exposed / pd (1)	log	0,0019802
QRP / quinolones	none	courses / adm (1)	identity	0,0015743	exposed / patients	identity	0,0015791	exposed / adm (1)	identity	0,0033871

Note: 3GC: third-generation cephalosporins; adm: admissions; ARC: aminoglycoside-resistant coliforms; CREKP: carbapenem-resistant *E. coli* , *Klebsiella* sp. and *Proteus* sp.; CRP: carbapenem-resistant Pseudomonas sp.; ICU: intensive care unit; MRSA: methicillin-resistant *Staphylococcus aureus* ; pd: patient-days; PTRC: piperacillin-tazobactam-resistant coliforms; PTRP: piperacillin-tazobactam-resistant Pseudomonas sp.; QRC: quinolone-resistant coliforms; QRP: quinolone-resistant Pseudomonas sp
