# Supplementary material for: Predicting Antimicrobial Resistance Prevalence and Incidence from Indicators of Antimicrobial Use: What Is the Most Accurate Indicator for Surveillance in Intensive Care Units?
Source: PLoS One. 2015 Dec 28;10(12):e0145088. doi: 10.1371/journal.pone.0145088 (PMC4692550; doi:10.1371/journal.pone.0145088)
Supplement: S4 Table — (PDF) [file pone.0145088.s004.pdf]

S2 Table. Most accurate, second most accurate and least accurate indicators in predicting incidence rates of antimicrobial resistance, for different scenarios, with their regression link and their mean absolute error, stratified per ICU type as adjusted for in regression models.

Resistance / antimicrobial use	Adjustment for ICU type	Most accurate indicator			Second most accurate indicator			Least accurate indicator		
		Indicator	Regression link	MAE	Indicator	Regression link	MAE	Indicator	Regression link	MAE
ARC / aminoglycosides	none	DDD / admissions	identity	0,0005978	courses / patient-days	identity	0,0005979	agent-days / admissions (1)	log	0,0006222
CREKP / 3GC + aminoglycosides + quinolones	adult and pediatric	DDD / patients	identity	0,0004915	DDD / admissions	identity	0,0004911	exposed / patient-days (1)	log	0,0005466
	neonatal	DDD / patients	identity	0,0001580	DDD / admissions	identity	0,0001590	exposed / patient-days (1)	log	0,0001691
CREKP / carbapenems	adult and pediatric	agent-days / patient-days	identity	0,0004366	courses / patient-days	identity	0,0004449	RDD / patients (1)	identity	0,0005497
	neonatal	agent-days / patient-days	identity	0,0001685	courses / patient-days	identity	0,0001663	RDD / patients (1)	identity	0,0001649
CRP / carbapenems	adult	courses / patient-days	log	0,0013694	DDD / patient-days	log	0,0013828	courses / patient-days (1)	log	0,0015481
	pediatric	courses / patient-days	log	0,0002591	DDD / patient-days	log	0,0002550	courses / patient-days (1)	log	0,0002780
	neonatal	courses / patient-days	log	0,0001448	DDD / patient-days	log	0,0001408	courses / patient-days (1)	log	0,0001421
MRSA / 3GC + penicillins + quinolones	adult	exposed / admissions (1)	log	0,0013244	exposed / patient (1)	log	0,0013315	exposed / patient-days	identity	0,0016956
	pediatric	exposed / admissions (1)	log	0,0010609	exposed / patient (1)	log	0,0010601	exposed / patient-days	identity	0,0011279
	neonatal	exposed / admissions (1)	log	0,0001088	exposed / patient (1)	log	0,0001085	exposed / patient-days	identity	0,0001075
MRSA / penicillins	adult	agent-days / patients (1)	log	0,0015978	courses / patients (1)	log	0,0015922	courses / patient-days	identity	0,0016926
	pediatric	agent-days / patients (1)	log	0,0010689	courses / patients (1)	log	0,0010832	courses / patient-days	identity	0,0011297
	neonatal	agent-days / patients (1)	log	0,0000995	courses / patients (1)	log	0,0001074	courses / patient-days	identity	0,0001070
PTRC / piperacillin-tazobactam	adult and pediatric	RDD / patients (1)	identity	0,0013841	DDD / patients (1)	identity	0,0013886	agent-days / admissions (1)	log	0,0015674
	neonatal	RDD / patients (1)	identity	0,0006871	DDD / patients (1)	identity	0,0006907	agent-days / admissions (1)	log	0,0006740
PTRP / piperacillin-tazobactam	adult and pediatric	agent-days / patients	log	0,0006989	agent-days / admissions	log	0,0007130	courses / patient-days (1)	log	0,0007643
	neonatal	agent-days / patients	log	0,0001984	agent-days / admissions	log	0,0001847	courses / patient-days (1)	log	0,0001977
QRC / quinolones	adult	courses / admissions	log	0,0013684	courses / patients	log	0,0013768	exposed / patient-days (1)	log	0,0015263
	pediatric	courses / admissions	log	0,0001665	courses / patients	log	0,0001665	exposed / patient-days (1)	log	0,0001709
	neonatal	courses / admissions	log	0,0001251	courses / patients	log	0,0001251	exposed / patient-days (1)	log	0,0001272
QRP / quinolones	none	DDD / admissions	identity	0,0003488	DDD / patients	identity	0,0003527	exposed / patient-days	log	0,0004326

Note: 3GC: third-generation cephalosporins; ARC: aminoglycoside-resistant coliforms; CREKP: carbapenem-resistant *E. coli*, *Klebsiella* sp. and *Proteus* sp.; CRP: carbapenem-resistant *Pseudomonas* sp.; ICU: intensive care unit; MRSA: methicillin-resistant *Staphylococcus aureus*; PTRC: piperacillin-tazobactam-resistant coliforms; PTRP: piperacillin-tazobactam-resistant *Pseudomonas* sp.; QRC: quinolone-resistant coliforms; QRP: quinolone-resistant Pseudomonas sp.
